# Evaluation of the New York City Green Carts program

**DOI:** 10.3934/publichealth.2015.4.906

**Published:** 2015-12-24

**Authors:** Shannon M Farley, Rachel Sacks, Rachel Dannefer, Michael Johns, Margaret Leggat, Sungwoo Lim, Kevin Konty, Cathy Nonas

**Affiliations:** 1 Bureau of Chronic Disease Prevention and Tobacco Control, New York City Department of Health and Mental Hygiene; 2 Bureau of Epidemiology Services, New York City Department of Health and Mental Hygiene; 3 Division of Epidemiology, New York City Department of Health and Mental Hygiene; 4 Center for Health Equity, New York City Department of Health and Mental Hygiene

**Keywords:** evaluation, food access, fruits and vegetables, nutrition, low income

## Abstract

Access to fresh fruits and vegetables is a concern, particularly among low-income populations. Mobile vending is one strategy to expand produce availability and access to increase consumption. In 2008, New York City launched a mobile vending initiative, Green Carts. We report on the evaluation. Three waves of cross-sectional observational surveys of produce availability, variety, and quality were conducted during the summers of 2008, 2009, and 2011 in a stratified random sample of stores and carts comparing establishments in Green Cart neighborhoods (n = 13) with comparison neighborhoods (n = 3). Bivariate analyses for availability, variety, and quality comparing Green Cart and comparison neighborhoods were presented across years, and logistic and negative binomial regressions were used to test whether fruit and vegetable availability, variety, and quality increased in Green Cart compared with comparison neighborhoods, adjusting for clustering and neighborhood demographics. Establishments selling fruits and vegetables in Green Cart neighborhoods increased between 2008 and 2011 (50% to 69%, *p* <0.0001); there was no comparable increase in comparison neighborhoods. Establishments selling more than 10 fruits and vegetables types increased from 31% to 38% (*p* = 0.0414) in Green Cart neighborhoods; there was no change in comparison neighborhoods. Produce quality was high among comparison establishments, with 95% and 94% meeting the quality threshold in 2008 and 2011, while declining in Green Cart neighborhood establishments from 96% to 88% (*p* < 0.0001). Sustained produce availability was found in Green Cart neighborhoods between 2008–2011. Green Carts are one strategy contributing to improving produce access among New Yorkers.

## 1. Introduction

Access to fresh fruits and vegetables is both a national and local concern, as produce is an important component of a healthy diet. [1]–[4] In particular, substantial interest surrounds removing barriers to produce consumption among low-income populations, including contextual factors, such as features of neighborhood environments that may influence availability and access in different geographies. [5]–[8] For instance, federal funding sources, including the Centers for Disease Control and Prevention Communities Putting Prevention to Work program, the Healthy Food Financing Initiative, and the reauthorization of the Farm Bill in 2014 among many others, provide support to local programs to increase fruit and vegetable access, as well as other healthy foods, with a focus on lower income populations. [9]–[11] Additionally, states, local municipal authorities, and other community organizations have collaborated to improve produce access through various programs. Such programs include increasing the number of farmers' markets in low-income communities, providing coupons or vouchers for the purchase of additional produce at farmers' markets, supporting new construction or renovations of supermarkets, and encouraging mobile vending. [12]–[18]

Mobile vending has been identified as one promising strategy for improving access to fresh produce. [15] In New York State's Capital District Region, the Veggie Mobile program aimed to increase fruit and vegetable consumption among low-income seniors using a refrigerated box truck that makes 1-hour weekly stops at senior centers, public housing projects, and other densely populated locations throughout the district. [16] A significant increase in fresh vegetable consumption was documented (from 1.98 to 2.58 servings/day, *p* = 0.027), as well as a decrease in frequency of trips to the supermarket (from 32.9% to 9.5% reporting shopped more than twice per week, *p* = 0.001), demonstrating the potential of this low-cost intervention to increase fruit and vegetable consumption among low-income seniors. [16] In an intervention focused on Hispanic and African American schoolchildren and their after-school purchases in Oakland, California, researchers introduced mobile vendors, *frutero*s, selling pre-cut and–bagged fruit and vegetables near schools. [17] Children and adults bought more precut fruits and vegetables from these vendors even in the presence of other vendors selling non-nutritious options: *fruteros* sold one additional bag of produce each day compared with non-nutritious vendors who sold 1.5 fewer non-nutritious items each successive day. [17]

Using the social-ecological model, the New York City (NYC) Department of Health and Mental Hygiene (DOHMH) has developed a variety of programs to improve fruit and vegetable availability citywide, focusing on low income neighborhoods since 2005. These efforts include providing coupons for the purchase of fruit and vegetables, offering zoning and financial incentives to retain established supermarkets and attract new stores, and working directly with corner store owners to improve healthy food options in the neighborhoods of interest. [19] Mobile vending was added in 2008 through the Green Carts program, launched as one component of the broader social-ecological strategy to address neighborhood disparities in the availability and consumption of healthy foods in NYC and positively influence the local food environment. The NYC Administrative Code was amended to expand the number of mobile vendors that sell whole fresh fruits and vegetables in the NYC neighborhoods with the lowest consumption of fruits and vegetables. [19] The Green Carts program consisted of staggered provision of 1,000 new ‘Green Cart’ mobile vending permits. To participate in the program, vendors were required to obtain a license for mobile food vending as well as the Green Cart permit for the cart itself, [20] and had to provide their own cart, including Green Cart vendors as one group of small business owners in NYC.

Between 2008 and 2011, the DOHMH conducted an evaluation of the Green Carts program to determine whether neighborhood access to fruits and vegetables improved overall, and to assess any associated impacts on neighboring food retailers after implementation of Green Carts. We hypothesized that Green Carts neighborhood establishments would have improved availability, variety and quality of produce compared with comparison neighborhood establishments. This paper describes and reports our findings.

## 2. Methods

To measure the impact of the Green Cart program on availability of fresh fruits and vegetables, observational surveys were conducted in randomly selected establishments selling food located in selected Green Cart police precincts and non-Green Cart comparison police precincts. Police precincts were selected as designations for Green Cart boundaries to facilitate enforcement of appropriate use of permits. In these precincts, 14%–32% of residents surveyed in 2004 reported consuming no fruits and vegetables the previous day, compared with 0–13% in the rest of the city. [21] All data were collected during the summer, when the likelihood of mobile vending is high, and fruit and vegetable options are numerous. Baseline data were collected during July and August of 2008, before the first 500 Green Cart permits were available, and follow-up data were collected one year later, during the summer of 2009. The second set of 500 permits was made available in September of 2009 and the second round of follow-up data was collected in the summer of 2011.

### 2.1. Sample Design

**Figure 1.**
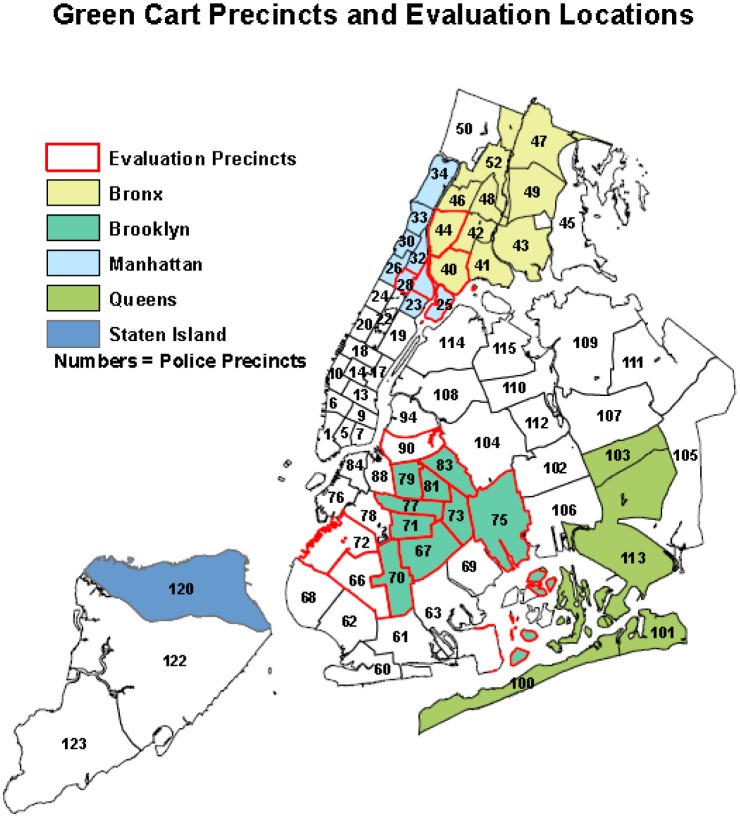
Green Cart precincts, DOHMH 2008.

Green Carts were permitted to sell in 32 out of 76 police precincts in NYC. (Figure 1) Vendors were assigned to boroughs and were permitted to sell in any designated Green Cart precinct (GCP) within their assigned borough. Due to limited resources, only a sample of precincts was included in the evaluation. Random selection of evaluation precincts was not possible because vendors were not required to identify in which precinct they plan to set-up their Green Cart within their assigned borough. To ensure that Green Carts were encountered during the evaluation, all eligible precincts in one entire borough, Brooklyn, were included in the sample. As vendors are not required to locate within specific precincts within their assigned boroughs, by sampling a full borough we were assured of capturing a sufficient number of Green Carts. To provide representation of other Green Cart precincts, we selected four GCPs from other high need areas (precinct 28 in Central Harlem, precinct 25 in East Harlem and precincts 40 and 44 in the South Bronx). Three non-Green Cart precincts, 66, 72, and 90, were selected as comparison precincts because they were the only non-Green Cart precincts with similar socio-demographic characteristics including income (GCP: $24,500 vs. Comparison: $28,900), race/ethnicity (GCP: 30.4% Hispanic vs. Comparison: 34.5% Hispanic), language spoken at home (GCP: 40.7% no English spoken at home vs. Comparison: 69.5% no English spoken at home), and nativity (GCP: 31.2% foreign-born vs. Comparison: 38.3% foreign-born), as the initiative neighborhoods.

A random sample of food retail stores was selected from the study precincts using New York State Department of Agriculture and Markets (NYSDAM) store lists in 2008, 2009, and 2011. NYSDAM keeps a list of retail stores that sell food, including their addresses and square footage. Stores were categorized into four types: supermarkets, small grocery stores, fruit and vegetable stores, and bodegas, based on listed store name and square footage. Bodegas are small corner stores that are ubiquitous throughout NYC, and serve as important sources of groceries in low-income neighborhoods. Stores that did not fit into these categories were excluded, including pharmacies, gas stations, specialty shops, vitamin stores, and bakeries, since they were not expected to carry fresh fruits and vegetables. A random sample was drawn from each eligible store type by precinct. The overall sampling rate was initially set at 25% of all eligible stores in the sampled precincts, based on overall store numbers for the selected precincts. Because there were more GCPs (13) than comparison precincts (3), the sample rate for stores in the comparison areas was increased to 37.5%, as comparison precincts had less stores overall to choose from, while the sampling rate was decreased to 20% in the GCPs, due to resource limitations. If a selected store was deemed ineligible upon the encounter, i.e., closed or a different store type, a new one was randomly chosen from the un-sampled vendors. Refusals by stores or carts to be surveyed were not replaced. Bodegas were not re-sampled due to high volume. The sample from 2008 was reused in 2009 and 2011 and replacements were randomly selected from the updated store list. Very few stores (less than 10 per year) were replaced.

Each summer of data collection, all precincts included in the study were canvassed on foot or by bus during business hours on weekdays in June when it was not raining to count the number of mobile fruit and vegetable carts on the streets. All carts found during canvassing were included in the sample and were surveyed. There were three types of carts: traditional, non-traditional, and Green Carts. Traditional carts were vendors with metal carts on wheels selling fruits and vegetables; non-traditional carts included vendors selling fruits and vegetables out of a van, setting up a folding table on the street, or using a shopping cart as the point of sale; and Green Carts were similar to traditional carts, with vendors using metal carts on wheels selling whole fruits and vegetables with a borough-specific Green Cart permit and the distinctive Green Cart umbrella. This procedure led to a sample of 422 establishments in the GCPs and 230 establishments in the comparison precincts for baseline data collection, 474 establishments in GCPs and 235 in comparison precincts for the year one follow-up, and 472 establishments in GCPs and 240 in comparison precincts for the year two follow-up. The fluctuations in sample size were due to changes in the number of carts (65 carts in 2008 versus 126 in 2009 and 122 in 2011).

### 2.2. Measures

Fruit and vegetable observations were measured using an instrument developed for palm pilots by Morland et al. (2007) to document the types, quality, and quantity of produce available in an establishment. The original questionnaire was modified for this study. [22] The instrument documented the type of establishment (supermarket, small grocery store, fruit and vegetable store, bodega, Green Cart, or other cart) and whether fruits and vegetables were for sale. The fruits and vegetables available at each establishment were recorded, including type (e.g., apple) and variety (e.g., Red Delicious, Golden Delicious, Gala, Granny Smith, Macintosh, etc.). Space was provided to record produce types and varieties not listed on the form. Data collectors had a glossary with color photos to aid in identifying produce varieties with which they were not familiar. The number of produce varieties per store was summed and stores were identified as carrying 1–5, 6–10 and more than 10 produce varieties. Data collectors rated the quality of each type of fruit and vegetable available as poor, average, or great. All data collectors were trained using the same guidelines for assessing quality in a one-day classroom and off-site training that included practicing with the survey tool in the classroom and at multiple stores and carts. First, the data collection team assessed a store together, compared findings and resolved discrepancies. The team then assessed multiple stores and carts with each person completing the assessment individually, and comparing results at the end of the day to ensure reliability across data collectors. The percentage of all produce items that were rated as ‘average’ or ‘great’ served as an acceptable threshold of produce quality for an establishment, and responses were collapsed together for analyses.

Availability was operationalized as the proportion of stores with no fruits or vegetables, only fruit, only vegetables, and fruits and vegetables. Variety was operationalized as the total number of unique types of fruits and vegetables available in each establishment. Quality was measured by averaging the percent of average/great ratings of the produce available overall in each establishment.

### 2.3. Analyses

Availability, variety, and quality were reported for both Green Cart and comparison neighborhoods across all years, among all establishments and among only bodegas, reporting percents and 95% confidence intervals. Trends over time were assessed using a chi-square test for availability data, a t-test for variety data, and a linear regression Wald F-test for quality data.

Generalized estimating equations were used to develop logistic and negative binomial regression models to test whether fruit and vegetable availability, variety, and quality increased in GCPs compared with comparison precincts over time (2008 vs. 2009 and 2011), while accounting for clustering effects. Models controlled for the proportion of income, race, nativity, and language spoken at home in each precinct based on data from the 2000 Census, as well as establishment type and the percentage of residents who had not consumed a fresh fruit or vegetable the previous day as reported in the 2008 NYC Community Health Survey. Logistic regression was used to examine three different models predicting establishments having no fruit and vegetables, having fruit and vegetables, and having fruit and vegetable variety of 10 or more, overall (Table 2) and among bodegas only (Table 3). Negative binomial regression examined models predicting establishments having average or great quality produce, overall (Table 2) and among bodegas only (Table 3). Main effects models were tested first (data not shown), followed by models that included a term representing the interaction between year and precinct type. The data were weighted to adjust for non-response and for varying probabilities of selection. Weights were calculated based on precinct and store type within Green Cart or comparison precincts. Weights were scaled so that the sum of the weights was equal to the total sample size. All analyses were performed in SAS 9.2 using proc survey procedures.

## 3. Results

### 3.1. Response rates

Response rates were generally high for all waves of the survey, with an 81% response rate in comparison precincts and an 87% response rate in GCPs for 2008, 88% and 83% respectively in 2009, and 89% and 94% in 2011.

### 3.2. Cart canvassing

In 2008, 51 carts were found in GCPs and 14 in comparison precincts. GCPs had 35 traditional carts and 16 non-traditional carts, and no Green Carts, as permits were not available yet, while comparison precincts had 12 traditional carts and 2 non-traditional carts. In 2009, 106 fruit and vegetable carts were identified in the GCPs and 20 carts in the comparison precincts. GCPs had 27 Green Carts, 10 traditional carts, and 69 non-traditional carts, while comparison precincts had 12 traditional carts and 8 non-traditional carts. In 2011, 97 fruit and vegetable carts were identified in the GCPs and 20 carts in the comparison precincts. GCPs had 67 Green Carts, 5 traditional carts, and 25 non-traditional carts, while comparison precincts had 18 traditional carts and 2 non-traditional carts.

### 3.3. Availability

In 2008, 50% of establishments in GCPs and 57% in comparison precincts sold both fruits and vegetables. (Table 1) The proportion of establishments selling both fruits and vegetables in GCPs increased significantly from 50% in 2008 to 68% in 2009 and 69% in 2011 (*p* <0.0001), whereas there were no statistically significant increases in comparison precincts between 2008 and 2011. This increase appeared to be driven by bodegas, as the proportion of bodegas in GCPs selling both fruits and vegetables increased from 45% to 65% (*p* <0.0001) while the increase in other establishments was smaller, from 83% to 92% (*p* = 0.03), compared with no significant change in comparison precinct bodegas (52% to 59%, *p* = 0.40) or other establishments (90% to 88%, *p* = 0.38).

The first overall model (Table 2) found a decreasing trend in the proportion of GCP establishments with no fruits or vegetables over time, which was significantly different from comparison precincts after controlling for confounders (*p* = 0.002; interaction of year and precinct). The bodega model (Table 3) showed large declines in stores without fruits and vegetables over time in GCPs compared with comparison precinct bodegas (*p* = 0.003; interaction of year and precinct).

The proportion of establishments in GCPs selling both fruits and vegetables increased significantly over time compared to comparison precincts after controlling for confounders (*p* <0.0001; interaction of year and precinct). Bodegas in GCPs also demonstrated significantly greater increases in the proportion of establishments selling both fruits and vegetables over time than did bodegas in comparison precincts (*p* <0.0001; interaction of year and precinct).

### 3.4. Variety

The number of establishments in GCPs selling more than 10 types of fruits and vegetables increased from 31% to 38% (*p* = 0.04) between 2008 and 2011, whereas there was no significant change in comparison precincts (52% and 47%, *p* = 0.37). All Green Carts carried more than 10 types of fruits and vegetables in 2009 compared with 80% in 2011 (data not shown).

The variety of fruits and vegetables in GCP establishments increased, while no change was observed in comparison precincts, and testing for the interaction of precinct and time found that GCPs were more likely to increase the proportion of establishments selling more than 10 fruits and vegetables compared with comparison precincts (*p* <0.0004; interaction of year and precinct), with the same pattern occurring in bodegas (*p* <0.0004; interaction of year and precinct).

### 3.5. Quality

Produce quality remained consistently high among comparison precinct establishments, with 95% and 94% of meeting the quality threshold, while declining in GCP establishments from 96% to 88% (*p* <0.0001).

While the overall proportion of fruits and vegetables rated as “average” or “great” was high, there was a decline in quality among GCP establishments compared with comparison establishments (*p* = 0.02; interaction of year and precinct). Bodegas had the lowest quality scores compared with other establishments combined (94% vs. 98%, *p* <0.0001, data not shown) and while the quality of produce in bodegas declined, there was no interaction of precinct and time for bodega quality.

**Table 1.** Green Cart Evaluation Availability Survey Baseline, and Follow-ups 1 and 2 unadjusted trends, 2008, 2009 and 2011.

	**Green Cart neighborhoods**				**Comparison neighborhoods**			
	**2008**	**2009**	**2011**	**GC *X*^2^, t-test, and F-test trend**	**2008**	**2009**	**2011**	**C *X*^2^, t-test, and F-test trend**
**Availability**	**% (95% CI)**	**% (95% CI)**	**% (95% CI)**	**p-value**	**% (95% CI)**	**% (95% CI)**	**% (95% CI)**	**p-value**
No fruit or vegetables	30 (25, 35)	19 (15, 24)	15 (11, 19)	< 0.0001	32 (26, 39)	21 (15, 27)	26 (20, 32)	0.0579
Fruit and vegetables	50 (45, 55)	68 (63, 73)	69 (64, 74)	< 0.0001	57 (50, 64)	65 (58, 71)	64 (57, 71)	0.2674
Just fruit	10 (7, 13)	9 (6, 11)	9 (6, 12)	0.7419	6 (3, 10)	7 (3, 11)	7 (4, 11)	0.9149
Just vegetables	10 (7, 14)	4 (2, 6)	7 (4, 9)	0.0103	4 (1, 8)	7 (3, 11)	3 (1, 6)	0.2043
**Variety**								
1–5 types of fruit or vegetables	44 (38, 50)	35 (30, 41)	38 (32, 43)	0.1167	28 (20, 36)	27 (20, 34)	23 (16, 30)	0.3584
6–10 types of fruit or vegetables	26 (20, 31)	25 (20, 30)	24 (19, 29)	0.6732	20 (13, 27)	23 (16, 29)	30 (22, 37)	0.0488
10+ types of fruit or vegetables	30 (25, 36)	40 (34, 45)	38 (33, 43)	0.0414	52 (44, 61)	50 (42, 58)	47 (39, 55)	0.3697
Quality								
“Average” or “great” quality	96 (94, 98)	96 (95, 98)	88 (86, 91)	<.0001	94 (92, 97)	95 (94, 97)	94 (92, 97)	0.9521
**Bodegas Only**								
	**Green Cart neighborhoods**				**Comparison neighborhoods**			
	**2008**	**2009**	**2011**	**GC trend**	**2008**	**2009**	**2011**	**C trend**
**Availability**	**% (95% CI)**	**% (95% CI)**	**% (95% CI)**	**p-value**	**% (95% CI)**	**% (95% CI)**	**% (95% CI)**	**p-value**
No fruit or vegetables	34 (28, 39)	23 (18, 28)	18 (14, 23)	0.0002	37 (29, 44)	26 (19, 33)	31 (24, 39)	0.1191
Fruit and vegetables	45 (39, 51)	65 (59, 71)	65 (59, 70)	< 0.0001	52 (44, 60)	59 (51, 66)	59 (51, 67)	0.4019
Just fruit	10 (6, 13)	8 (4, 11)	9 (6, 12)	0.6696	7 (3, 11)	7 (3, 11)	6 (2, 10)	0.9304
Just vegetables	11 (8, 15)	5 (2, 7)	8 (5, 11)	0.0127	5 (1, 8)	9 (4, 13)	4 (1, 7)	0.2016
**Variety**								
1–5 types of fruit or vegetables	51 (44, 58)	40 (33, 47)	46 (39, 53)	0.3163	33 (24, 43)	33 (24, 41)	26 (18, 35)	0.3522
6–10 types of fruit or vegetables	29 (23, 36)	30 (24, 37)	29 (23, 35)	0.9340	22 (14, 31)	27 (19, 36)	37 (27, 46)	0.0318
10+ types of fruit or vegetables	20 (14, 26)	30 (24, 36)	25 (20, 31)	0.1992	44 (34, 54)	40 (31, 49)	37 (27, 46)	0.2616
**Quality**								
“Average” or “great” quality	96 (94, 98)	96 (94, 97)	86 (83, 89)	<0 .0001	94 (91, 96)	95 (92, 97)	93 (90, 96)	0.7696

Note: Statistical test chi-square was used for availability, a t-test for variety, and a linear regression Wald F-test for quality data.

**Table 2.** Green Cart Evaluation Availability Survey Baseline, and Follow-ups 1 and 2 overall adjusted trends and interaction.

	No F&V model		F and V model		Variety 10+ model		Quality model	
	All stores		All stores		All stores		All stores	
	OR (95% CIs)	Chi-sqp-value	OR (95% CIs)	Chi-sqp-value	OR (95% CIs)	Chi-sqp-value	OR (95% CIs)	Chi-sqp-value
**Year**								
2008	1.00		1.00		1.00		1.00	
2009	0.75 (0.65, 0.86)	**< 0.0001**	1.02 (0.76, 1.38)	0.8789	1.28 (1.03, 1.60)	**0.0247**	0.76 (0.70, 0.83)	**< 0.0001**
2011	0.57 (0.37, 0.88)	**0.0013**	1.34 (0.93, 1.95)	0.1195	0.82 (0.76, 0.89)	**< 0.0001**	1.16 (0.99, 1.35)	0.0657
**Precincts**								
Comparison	1.00		1.00		1.00		1.00	
Green Cart	0.61 (0.14, 2.57)	0.4989	0.84 (0.26, 2.69)	0.7709	1.00 (0.23, 4.31)	0.9981	0.98 (0.85, 1.13)	0.7475
**Store type**								
Bodega	1.00		1.00		1.00		1.00	
Supermarket	32.41 (2.71, 388.24)	**0.0060**	3.17 (0.89, 11.26)	0.0739	2.70 (1.09, 6.71)	**0.0322**	1.08 (1.03, 1.14)	**0.0038**
Small grocery	17.24 (1.75, 169.78)	**0.0147**	0.11 (0.02, 0.59)	**0.0104**	0.05 (0.01, 0.31)	**0.0018**	1.06 (1.00, 1.13)	0.0564
F&V vendors	0.19 (0.06, 0.61)	**0.0051**	7.77 (2.89, 20.84)	**< 0.0001**	21.38 (11.18, 40.90)	**<.0001**	0.93 (0.89, 0.97)	**0.0011**
**Interaction**								
Year*GCvC	0.56 (0.39, 0.81)	**0.0021**	2.24 (1.53, 3.28)	**< 0.0001**	1.83 (1.31, 2.55)	**0.0004**	1.11 (1.01, 1.21)	**0.0228**

Models controlled for the proportion of income, race, nativity, language spoken at home, and population that did not eat any fruits and vegetables the day before in each precinct based on data from the 2000 Census and the 2008 CHS.

**Table 3.** Green Cart Evaluation Availability Survey Baseline, and Follow-ups 1 and 2 adjusted trends among bodegas and interaction.

	No F&V model		F and V model		Variety 10+ model		Quality model	
	Bodegas		Bodegas		Bodegas		Bodegas	
	OR (95% CIs)	Chi-sqp-value	OR (95% CIs)	Chi-sqp-value	OR (95% CIs)	Chi-sqp-value	OR (95% CIs)	Chi-sqp-value
**Year**								
2008	1.00		1.00		1.00		1.00	
2009	0.75 (0.65, 0.86)	**< 0.0001**	0.99 (0.73, 1.34)	0.9352	1.21 (1.00, 1.47)	**0.0489**	0.84 (0.81, 0.88)	**< 0.0001**
2011	0.59 (0.38, 0.92)	**0.0197**	1.31 (0.89, 1.93)	0.1692	0.79 (0.72, 0.86)	**< 0.0001**	0.93 (0.88, 0.97)	**0.0006**
**Precincts**								
Comparison	1.00		1.00		1.00		1.00	
Green Cart	0.60 (0.14, 2.52)	0.4869	0.85 (0.25, 2.95)	0.7976	0.91 (0.21, 4.00)	0.8992	0.83 (0.63, 1.08)	0.1586
**Interaction**								
Year*GCvC	0.57 (0.40, 0.83)	**0.0029**	2.29 (1.53, 3.43)	**<.0001**	1.89 (1.32, 2.72)	**0.0006**	0.97 (0.90, 1.06)	0.5437

Models controlled for the proportion of income, race, nativity, language spoken at home, and population that did not eat any fruits and vegetables the day before in each precinct based on data from the 2000 Census and the 2008 CHS.

## 4. Discussion

Between 2008 and 2011, the availability and variety of fruits and vegetables in GCPs increased compared with non-Green Cart precincts. GCP establishments also demonstrated increases in the proportion of establishments carrying more than 10 types of fruits and vegetables, while there was no change in comparison precincts. While fruit and vegetable quality in comparison precincts remained constant, there was a significant decline in quality among GCP establishments. Most of these changes were driven by bodegas. Thus, while the increased number of Green Carts expanded produce availability, this evaluation indicates that their presence may have also encouraged other establishments, particularly bodegas, to sell more produce.

An earlier assessment of Green Carts in the Bronx by Lucan et al. (2011) found that Green Carts tended to cluster around health centers, transportation hubs, and business districts. [23] The authors suggested that the carts may not have extended the accessibility of fresh produce to the residential neighborhoods most in need, thereby threatening program viability due to increased competition among vendors. Another study critiquing the Green Carts program found that Green Carts were not alleviating food deserts in NYC. [24] These viewpoints were contrary to the idea of a free market business, which demonstrated that co-locating Green Carts in highly trafficked areas increased the profitability of these new businesses and therefore contributed to the program's viability. [25] Clustering of all types of carts is common in NYC, and clustered carts provide convenient access to reasonably priced produce. While the DOHMH provides permits, Green Cart vendors are independent businesses that make their own decisions about cart location, variety of produce, operating hours, and access to produce. A previous report concluded that keys to success for Green Cart vendors include: 1) setting up in a good location; 2) maintaining an attractive and diverse cart; 3) having a vendor that speaks the same language as the community residents; and 4) having the support of one or several nearby community organizations. [25] Additionally, as in farmers' markets, electronic benefit terminals (EBT) are now available for Green Cart vendors who qualify.

The quality of fruits and vegetables declined overtime among GCP establishments, particularly bodegas, without a similar decline in comparison precincts; however, in interaction models, only the overall trend was significant, and not the trend for bodegas, possibly demonstrating that when adjusting for socio-demographics, there were no differences in quality across GCP and comparison precincts over time among bodegas. While it is possible that Green Carts affected bodega produce sales, Dannefer et al. (2015) have shown that most people purchase produce at supermarkets, and only a small proportion at bodegas or carts, including Green Carts. [26] Another possibility is that with the influx of Green Carts and other fruit and vegetable promotion activities and programs, bodegas may have expanded their produce selection but not experienced a parallel uptake in sales, resulting in produce remaining on shelves longer and quality declining. [19]

This study has some limitations. Improvements cannot be solely attributed to Green Carts, as other DOHMH programs and outside factors may have also contributed to findings of this evaluation. It is not possible to know to what extent the Green Cart program motivated bodegas to improve their selection of produce. Identifying comparison precincts that were similar to GCPs resulted in selection of all comparison precincts from Brooklyn. Therefore, to control for differences between precincts within boroughs, estimates of the Green Cart effect were adjusted for precinct-level characteristics such as racial/ethnic composition, nativity and income. Quality was a subjectively measured item that may have been affected by the day of week and time of day when stores were visited, as well as by differences in data collectors. We used proportions to assess how the presence of Green Carts contributes to availability of produce in a neighborhood. This helps adjust for the varying size of the neighborhood as well as the variation in response rate over time, in addition to larger retailer trends. Proportions are a coarse measure of change and provide a conservative test of the contributions Green Carts made to produce availability. Additionally, data were only collected during the summer months and does not represent shifts in year-round produce availability and variety.

Overall, the results show an increase in sustained fruit and vegetable availability, most likely influenced by an increase in both fruit and vegetable availability in bodegas in GCPs from 2008 to 2011. This increase is not likely due to a secular trend, as these same patterns were not found in our comparison group. However, these results may not represent the full potential of the Green Cart vendors. Though half of the Green Cart permits were distributed during the 2011 data collection period, many Green Cart vendors were not actively vending at the time of data collection. In the 13 Green Cart precincts canvassed for the evaluation, only 67 Green Carts were found. Green Carts have no set time or days to vend, so it is possible that there were others not seen by data collectors. Additionally, there were multiple Health Department interventions in areas that overlapped with GCPs and the results likely reflect a combination of initiatives by the DOHMH. Media campaigns and news stories about Green Carts may also have affected fruit and vegetable awareness in the community, in addition to the carts themselves. Between 2002 and 2012, the prevalence of NYC adults reporting no consumption of fruits and vegetables the previous day declined from 14.3% to 12.5% (*p* <0.001), indicating that consumption is improving at the population level, which may be in part due to Green Carts. [19] In sum, Green Carts have emerged as one successful strategy among a constellation of initiatives introduced by the DOHMH to increase produce consumption among New Yorkers.
